# Understanding the determinants of the complex interplay between cost-effectiveness and equitable impact in maternal and child mortality reduction

**DOI:** 10.7189/jogh.02.010406

**Published:** 2012-06

**Authors:** Mickey Chopra, Harry Campbell, Igor Rudan

**Affiliations:** 1 UNICEF, New York, USA; 2 Centre for Population Health Sciences and Global Health Academy, The University of Edinburgh Medical School, Edinburgh, Scotland, UK

## Abstract

**Background:**

One of the most unexpected outcomes arising from the efforts towards maternal and child mortality reduction is that all too often the objective success has been coupled with increased inequity in the population. The aim of this study is to analyze the determinants of the complex interplay between cost-effectiveness and equity and suggest strategies that will promote an impact on mortality that reduce population child health inequities.

**Methods:**

We developed a conceptual framework that exposes the nature of the links between the five key determinants that need to be taken into account when planning equitable impact. These determinants are: (i) efficiency of intervention scale-up (requires knowledge of differential increase in cost of intervention scale-up by equity strata in the population); (ii) effectiveness of intervention (requires understanding of differential effectiveness of interventions by equity strata in the population); (iii) the impact on mortality (requires knowledge of differential mortality levels by equity strata, and understanding the differences in cause composition of overall mortality in different equity strata); (iv) cost-effectiveness (compares the initial cost and the resulting impact on mortality); (v) equity structure of the population. The framework is presented visually as a four-quadrant graph.

**Results:**

We use the proposed framework to demonstrate why the relationship between cost-effectiveness and equitable impact of an intervention cannot be intuitively predicted or easily planned. The relationships between the five determinants are complex, often nonlinear, context-specific and intervention-specific. We demonstrate that there will be instances when an equity-promoting approach, ie, trying to reach for the poorest and excluded in the population with health interventions, will also be the most cost-effective approach. However, there will be cases in which this will be entirely unfeasible, and where equity-neutral or even inequity-promoting approaches may be substantially more cost-effective. In those cases, investments into health system development among the poorest that would increase the quality and reduce the cost of intervention delivery would be required before intervention scale-up is planned.

**Conclusions:**

The relationships between the most important determinants of cost-effectiveness and equitable impact of health interventions used to reduce maternal and child mortality are highly complex, and the effect on equity cannot be predicted intuitively, or by using simple linear models.

In recent years, enormous efforts have been made to estimate the global burden of maternal and child mortality and identify the main causes, study the role of risk factors, assess the effectiveness of available interventions, and to track the coverage of those interventions in low and middle-income countries [1-10]. However, this large body of evidence has not been followed by the development of sufficiently simple and accurate tools and approaches that effectively translate the evidence and information into health policy decisions where this is most needed - at the national and sub-national level in low-resource settings. In the absence of evidence-based planning, it is not surprising that unexpected outcomes can arise from efforts towards maternal and child mortality reduction. One of the most perplexing outcomes is that all too often the objective success in mortality reduction has been coupled with an increased health inequity in the population [11].

To understand the roots of this problem, we should appreciate that policy makers at the national and sub-national level have limited resources for scaling up cost-effective health interventions in their populations. When planning the “best buys” for committing their resources in maternal and child health, they are faced with a very complex task. They need to choose between at least several dozen interventions that target neonates, infants, children and mothers, most of which have been proven to be cost-effective in many contexts [4,5,8,9]. They soon realize that it would take more than a simple calculation to decide on the most rational way to invest in health intervention scale up. Depending on the local and national context, the interplay between many important factors will affect both cost-effectiveness and the impact on equity for their chosen intervention scale-up programs. Neglect (or improper understanding) of these complexities can lead to decisions which result in maternal and child mortality reduction not being achieved in the most cost-effective way, or being associated with increases in health inequity within communities. The present set of tools does not sufficiently capture the full array of factors [12].

The aim of this study is to analyze the determinants of the complex interplay between cost-effectiveness and equity in maternal and child mortality reduction and suggest strategies that promote an impact on mortality that will reduce population child health inequities. To achieve this aim, we develop a transparent framework based on several key epidemiological concepts that can be used to support national-level decision making in health intervention prioritization. Using this framework, we try to expose the complex interplay among factors that influence both cost-effectiveness and equity in child and maternal mortality reduction and identify the key information needs for planning of equitable and cost-effective programs of health intervention scale-up.

## METHODS

### The cost of intervention scale-up in different equity strata

The first important determinant to consider is *the cost of intervention scale-up in different equity strata*. In our framework, we will divide any population of interest into 5 equity strata (quintiles), each comprising 20% of the population, where Q1 denotes the wealthiest quintile and Q5 the poorest. The cost of achieving complete coverage with any health intervention will clearly differ between the wealthiest (Q1) and the poorest (Q5) quintile, but there is remarkably little information available on the determinants of these costs in each quintile and the actual differences in cost of implementation. It is also clear that these differences between strata will be intervention-specific and also context-specific, rather than following any “standard”, predictable pattern. This means that, for some interventions, the costs may not increase dramatically (from the wealthiest to the poorest quintile) with increasing coverage. In fact, wherever the salary of health professionals is the main component of the cost, then it is possible to envisage circumstances in which, for some interventions, it may be even cheaper to cover the poorest quintile (eg, when there is a well-developed network of village health workers who can administer cheap antibiotic treatment) than the wealthiest quintile (where this depends on skilled medical doctors who have access to both cheap and more expensive antibiotics). However, there will also be many examples where complete intervention coverage will be more readily achieved among the wealthy Q1 than in the poorest Q5, where it may be almost impossible or even unfeasible to achieve.

**Figure 1:**
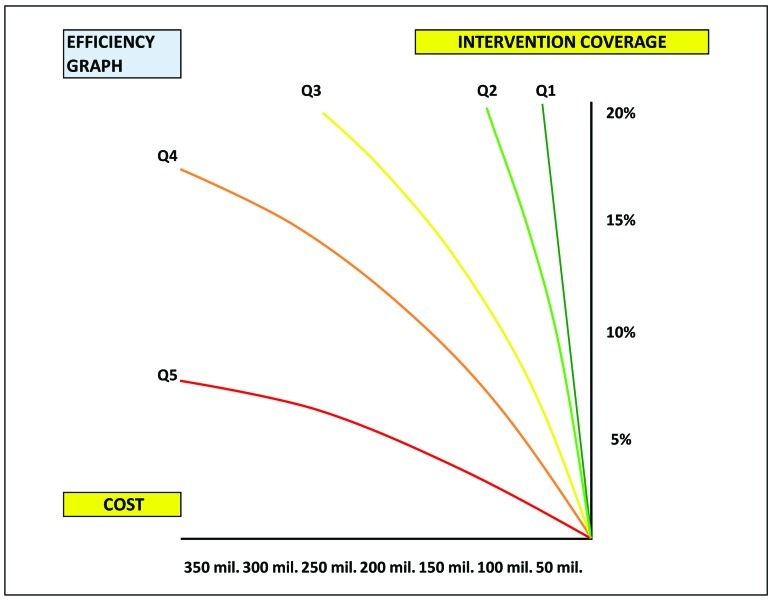
The relationship between cost of the intervention scale-up and achieved intervention coverage, which determines the efficiency of intervention delivery, presented for each of the five equity strata in the population (Q1 – the wealthiest quintile; Q5 – the poorest quintile).

**Figure 1** summarizes this relationship. The horizontal axis represents the increasing cost required for scaling up of an intervention, while the vertical axis measures the completeness of coverage in each equity quintile (ranging from 0% to 20% of the total population). Recently, substantial efforts have been made to track the coverage of interventions specifically by equity strata in many low and middle-income countries. This work has indicated that this is an important component that will be need to be included in planning the equitable delivery of interventions [13-15]. However, we still need information on the actual cost components of intervention scale-up and how these differ across wealth quintiles in varying contexts and for each intervention. In reality, this cost cannot be expressed as a fixed amount in US$ per person that is characteristic of each delivered intervention, nor does it increase linearly as the achieved population coverage increases (**Figure 1**). The cost of intervention scale-up includes more than just the market cost of an intervention (such as vaccine or a drug), because the successful delivery also requires everything else that is required to reach the targeted recipients, such as costs of health worker salaries, transport and storage, improved access and expanded outreach.

Those additional costs may be relatively small if the aim was to cover the most accessible 20% or 40% of mothers and children. In these circumstances the relationship between cost and achieved coverage may indeed be approximately linear. However, additional costs of intervention delivery will start increasing in complex and nonlinear patterns when the coverage of the most deprived children and mothers is attempted, because many obstacles need to be overcome to reach them. Because of these additional costs, all too often we observe that the most accessible mothers and children are being covered with ever more interventions, while the marginalized are missing out on all of them. This approach would still be expected to reduce maternal and child mortality, but the progress would be very slow and inequitable. This is because most child and maternal deaths occur among the most inaccessible parts of the population and only a minor part of the mortality burden is being targeted with interventions. The progress that is being achieved benefits only those who are accessible, thus increasing inequity. Reducing the additional costs of intervention delivery when targeting the poor would involve challenges that are related to both supply and demand for the prioritized interventions.

One examples of this relationship between cost and achieved intervention scale-up by equity quintiles (Q1-Q5) is shown in **Figure 1****.** This graph summarizes the *efficiency* of intervention delivery. In this hypothetical example, it is apparent that for the same intervention it is much cheaper, and therefore more efficient, to achieve full coverage in the most wealthy 20% of the population (Q1) than in the poorest quintile (Q5). In fact, in this example the difference in cost is so large that it poses a question whether the potential for mortality reduction in the poorest quintile (Q5) justifies such an inefficient delivery of a life-saving intervention at such a high cost? Sometimes, even when the equity argument is being respected, it may still be entirely unfeasible to attempt to reach the poorest Q5, because the infrastructure that would allow this in a cost-effective way simply does not exist. In such cases, investing in health system development may need to precede investing in intervention coverage. We will move through the rest of the framework to explore this further, because the answers will rarely be intuitive.

### The effectiveness of an intervention in different equity strata

The second determinant of cost-effectiveness and equity to consider is *the effectiveness of an intervention in different equity strata.* The effectiveness of an intervention, or its “potential impact fraction”, indicates which proportion of the current mortality burden that is targeted by an intervention would be averted among those who receive the intervention, in comparison to those that do not receive it. In theory, the effectiveness of an intervention in relation to a specific cause of death – such as a specific antibiotic treatment against childhood pneumonia – should be relatively similar in all settings. This is because it should primarily be determined by the biology of disease and the interplay between the disease and the intervention. However, the experience from the field tells us that the effectiveness of the same intervention may differ substantially between Q1 and Q5. Some of the reasons may be, in the above example, that there is different spectrum of pathogens among the very poor (and less well nourished) (Q5), and /or higher levels of antibiotic resistance, and/ or later presentation with more severe symptoms because of barriers in access to care or differences in care-seeking behaviour, all of which reduces the effectiveness of antibiotic treatment against pneumonia in comparison to Q1 children. In addition, and perhaps even more importantly, the quality of intervention delivery will not be the same in all socio-economic strata. Incomplete or inadequate delivery will be more likely among the poorest (Q5), which will decrease the effectiveness of the intervention against the same cause of death. Taking the example of pneumococcal and Hib vaccine against pneumonia, this may be because of more likely interruption of the cold chain when trying to reach the poorest, lower level of health workers' education and skills which may lead more often to inadequate administration of vaccines, and lower health awareness among the parents of the children leading to lower levels of full attendance for all immunization appointments.

**Figure 2:**
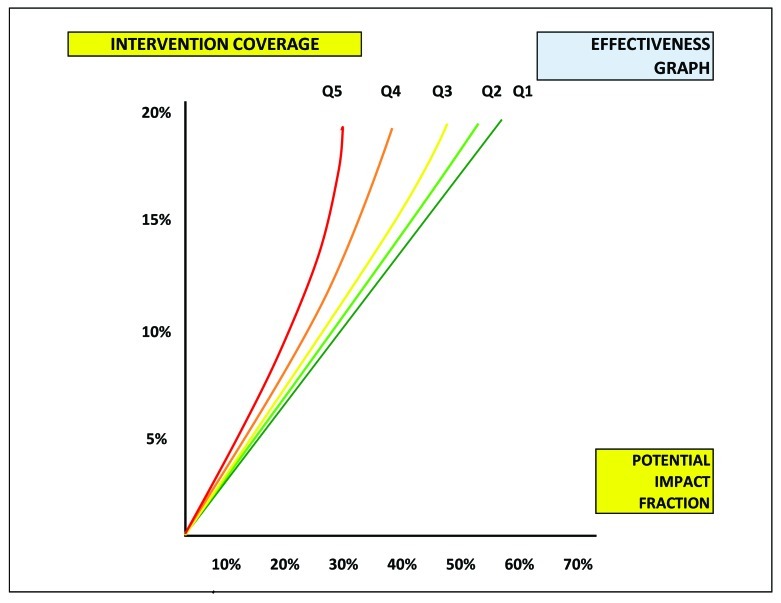
The relationship between achieved intervention coverage and potential impact fraction, which determines the effectiveness of the intervention, presented for each of the five equity strata in the population (Q1 – the wealthiest quintile; Q5 – the poorest quintile; to expose the continuum of relationships and effects that the important determinants in this framework have on mortality reduction and equity, the vertical axis is taken from Figure 1, while the horizontal axis measures the effectiveness in different equity strata.).

We tried to capture this complex relationship between the achieved coverage by equity quintiles and the effectiveness in mortality reduction in **Figure 2**. In order to expose the continuum of relationships and effects that the important determinants in this framework have on mortality reduction and equity, the vertical axis is taken from **Figure 1**. It again shows the achieved coverage by each equity quintile, which can range from 0 to 20%. The horizontal axis shows the effectiveness of the intervention of interest in terms of reduction in mortality in each equity stratum (expressed as a proportion of the total mortality in that stratum) that could be achieved for a given level of coverage shown on the vertical axis. The value on the horizontal axis where the coverage in Q1 becomes complete (in this case, between 50% and 60%) shows the maximum potential for the intervention to reduce mortality against a specific cause under ideal conditions. For example, if the cause of death of interest is pneumonia; if 50%-60% of pneumonia deaths in this setting are caused by *Pneumococcus*; and if pneumococcal vaccine is nearly 100% effective in preventing pneumococcal pneumonia deaths, then this is the maximum potential effectiveness of pneumococcal vaccine under ideal conditions. However, the adverse factors explained above (interruption of the cold chain, inadequate administration by health workers, failure to comply with full vaccination schedule by the parents) may act to reduce its effectiveness to only 20%-30% among the poorest section of the population in Q5, even when the full coverage is achieved (**Figure 2**). Presently, there is remarkably little understanding or evidence about the nature and scale of differences in effectiveness of health interventions in different equity strata, although this is one of the most important determinants of overall cost-effectiveness and equitable impact.

### The size and composition of the mortality burden in different equity strata

**Figure 3:**
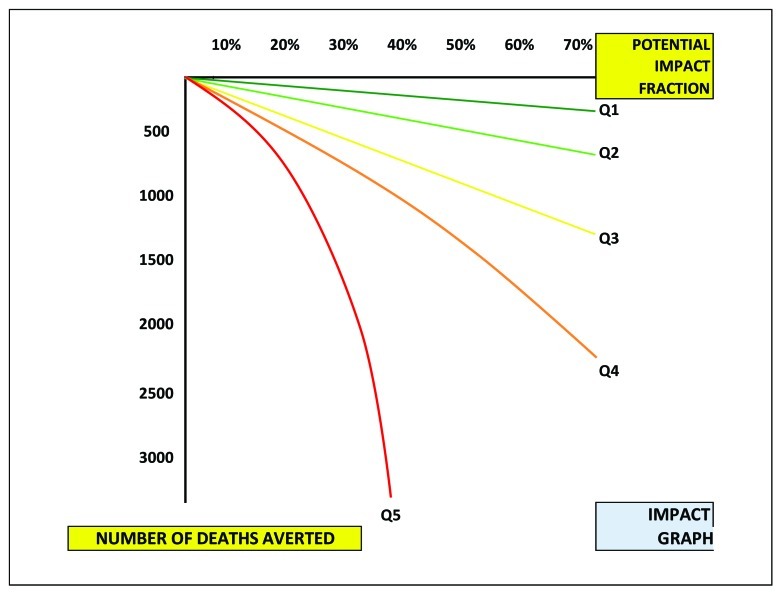
The relationship between potential impact fraction and number of deaths averted, which determines the potential impact of the intervention in mortality reduction, presented for each of the five equity strata in the population (Q1 – the wealthiest quintile; Q5 – the poorest quintile; to expose the continuum of relationships and effects that the important determinants in this framework have on mortality reduction and equity, the horizontal axis is taken from Figure 2, while the vertical axis measures the number of deaths that could potentially be averted in different equity strata.).

The third important determinant to consider is the *absolute size of the mortality burden in different equity strata and its composition*. The relationship between the burden of mortality and equity strata is rather predictable: the absolute number of deaths will always be much greater in the poorest (Q5) than in the wealthiest quintile (Q1), given that the quintiles are of the same size by definition (ie, 20% of population), and that mortality rates are greater among the poor. However, the graph that captures this relationship (**Figure 3**) may still look very differently, depending of the level of inequity in the population. The lines representing the five equity strata in this graph may be relatively close to each other such as in a situation where the burden of mortality is, in absolute terms, only 2 times greater in Q5 compared to Q1. However, these lines could also be far apart such as when the burden of mortality is 10 times greater in Q5 than in Q1. In a sense, **Figure 3** is a visualization of the level of inequity in a society when expressed as bearing the burden of mortality. A substantial effort has been invested in recent years to understand and explore the differences in mortality rates between the equity strata in low and middle-income countries [9,14,16,17].

There is another factor that adds complexity to the relationship between intervention effectiveness and number of deaths averted by equity strata, as shown in **Figure 3**. The breakdown of the overall number of deaths by cause of death may differ quite substantially between equity quintiles. For example, causes of deaths among the wealthiest children will be dominated by congenital abnormalities, preterm birth complications and accidents – ie, the problems that even well-functioning health system still can’t easily tackle effectively. However, the poorest children will mainly be expected to die from infectious causes, such as pneumonia, diarrhea, malaria and neonatal sepsis. As an example, the proportional contribution of pneumonia to all child deaths observed in a developing country would typically be around 10% in the wealthiest quintile of children rising to up to 40% among the poorest children [18]. This is why the “potential impact fraction” of an intervention that only targets pneumonia in reduction of the overall child mortality burden could be much larger in the poorest (Q5) than in the wealthiest quintile (Q1) despite lower quality of delivery in Q5 settings acting to reduce the intervention effectiveness.

The graph presented in **Figure 3** therefore exposes the *potential impact* of intervention delivery to reduce the burden of mortality in absolute terms. In the hypothetical case shown in **Figure 3**, it is apparent that for an intervention that targets eg, infectious causes, it is usually more effective to achieve full coverage in the poorest 20% of the population, regardless of the reduced effectiveness because of poorer quality of delivery. However, for interventions that target causes of deaths that are more prominent among the wealthiest, such as eg, congenital abnormalities, these relationships would be inverse. Similarly, if a cause of death is equally important in all 5 strata, then the effectiveness of an intervention would usually be greater among the wealthy, because the lower quality of delivery and increased barriers to access and care-seeking would reduce it among the poor.

### The cost-effectiveness of investing in different equity strata

**Figure 4:**
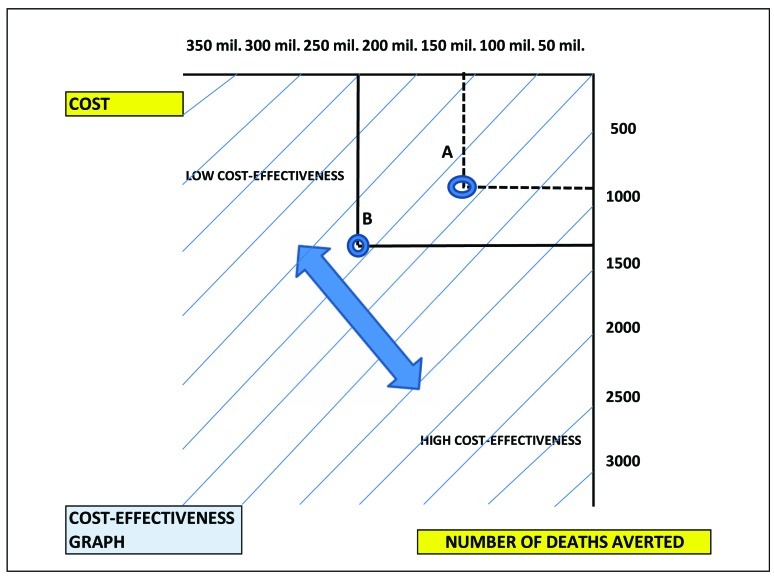
The relationship between the cost of intervention scale-up and number of deaths averted, which determines the cost-effectiveness of the intervention in mortality reduction, presented for each of the five equity strata in the population (Q1 – the wealthiest quintile; Q5 – the poorest quintile).

The fourth determinant to consider is the one that usually drives policy decisions: *the number of deaths averted per cost of intervention scale-up in different equity strata*. Health investors usually like to know how many deaths could be averted with a fixed level of investment. The more deaths averted per fixed investment, the more cost-effective the scale up. Therefore, **Figure 4** exposes the *cost-effectiveness* of many competing investment options.

**Figure 4** is drawn using the “cost” from **Figure 1** as a horizontal axis, and “the number of deaths averted” from **Figure 3** as the vertical axis. When the cost is low and the number of averted deaths high (ie, the bottom-right corner of **Figure 4**), the intervention scale-up is highly cost-effective. When the cost is high and the number of averted deaths low (ie, the top-left corner of **Figure 4**), the intervention scale-up is not cost-effective. In **Figure 4**, the hypothetical program that implemented intervention “A” proved to be more cost-effective than the program that implemented intervention “B”. However, the cost-effectiveness of mortality reduction does not necessarily mean that it will also be “equitable”, as these are two separate dimensions. Deaths can be reduced in a highly cost-effective way when investments are targeting the wealthiest quintiles, just as when they are targeting the poorest. In the former case, the mortality will be reduced, but the inequity will be increased. In the latter, both mortality and inequity will be reduced. We argue that this should be the goal whenever possible, and that a simple check using this framework can help highlight these important issues and enable decision-making that includes this goal. Scaling up health interventions in Q3 will be “equity-neutral”, scaling up in Q4 and Q5 will always be “equity-promoting”, while scaling up in Q1 and Q2 will be “inequity-promoting”; all three approaches, however, will result in reduction of mortality burden, and in some cases this reduction may even be more cost-effective when interventions are scaled in Q1 and/or Q2, rather than in Q4 an/or Q5.

### The complex interplay among factors that influence equity and cost-effectiveness of mortality reduction

**Figure 5:**
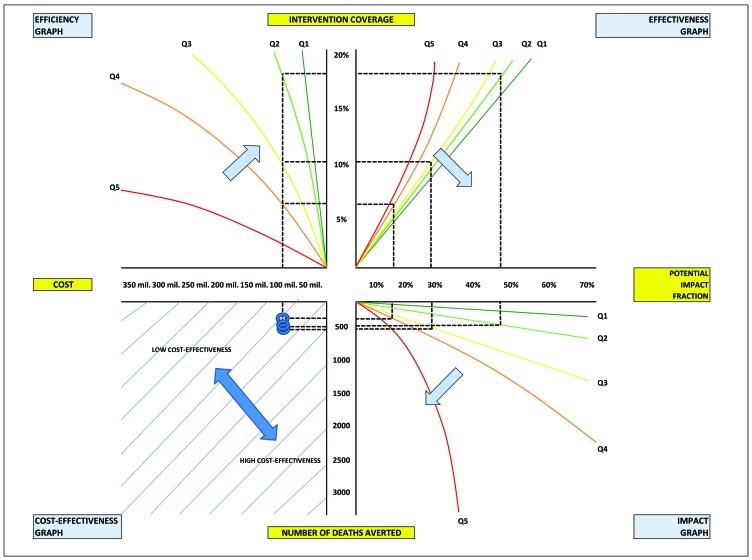
A hypothetical case of planning the delivery of a new intervention to different equity strata in the population (Q2 vs Q3 vs Q4) with a fixed budget and assessing its cost-effectiveness under equity-neutral (Q3), equity-promoting (Q4) or inequitable (Q2) strategy.

If we bring together the previous four graphs into a single decision-making framework, as shown in **Figure 5**, it becomes clear that the relationships between the four determinants (efficiency, effectiveness, impact on mortality and cost-effectiveness) and the impact on equity will not necessarily be intuitive in any setting. The final outcome will be governed by a series of complex and typically nonlinear relationships between the determinants above. Anything that increases the efficiency of delivery (see arrow in the top left quadrant, **Figure 5**), the quality of delivery (see arrow in the top right quadrant, **Figure 5**), and acts upon the greater mortality burden (see arrow in the bottom right quadrant, **Figure 5**) will be more cost-effective (see arrows in the bottom left quadrant, **Figure 5**), and vice versa. Increased efficiency and quality of delivery will tend to make scaling up among the wealthier groups more cost-effective, while the increased size of the burden will tend to make scaling up among the poorer groups more cost-effective (**Figure 5**).

To further illustrate the nature of this complexity, **Figure 5** offers an illustrative example: a fixed sum of money (shown on the “cost” axis) is available to ensure delivery of an entirely new intervention to children in a country. Local policy makers have a choice: if they assume that children in Q1 would find ways to get this intervention anyway, while those in Q5 are arguably too hard to reach, they could invest the available funds to cover as many children in Q2, Q3 or Q4 as possible. The difference is that covering Q2 would increase inequity, while covering Q4 would promote equity and Q3 would be equity-neutral. If similar cost-effectiveness between the three approaches could be demonstrated (in the bottom left quadrant of the proposed framework), then the equity-promoting approach (covering Q4) should be preferred. In this example, implementing the intervention to the children in Q3 is more cost-effective than the other two approaches (**Figure 5**), but the difference is not substantial and covering Q4 could be considered instead.

In the remainder of this paper, we will present and discuss a hypothetical case related to planning of the delivery of an intervention to different equity strata in the population and assessing its cost-effectiveness at different levels of investment.

## RESULTS

**Figure 6:**
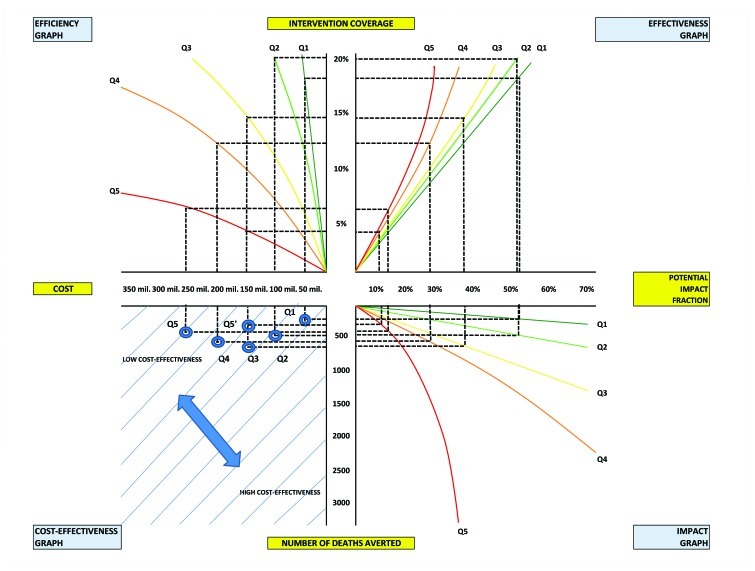
Six hypothetical investment cases of different amounts of funding for scale-up of the same intervention in 5 different equity strata, and with different level of investment into the poorest quintile (Q5).

We will consider a hypothetical case of framework implementation: planning of the delivery of a new intervention, such as vaccine, improved sanitation, or maternal education program, to different social strata in the population and assessing its cost-effectiveness. The upper left quadrant graph in **Figure 6** shows how the level of investment translates to intervention coverage in different equity quintiles in the population of interest (Q1 being the wealthiest and Q5 the poorest). Clearly, in the population of interest an investment of US$ 50 million will ensure nearly complete coverage of all 20% children in Q1 quintile, while complete coverage can be achieved with US$ 100 million in Q2. US$ 150 million will cover about 7 out of 10 children in Q3, while US$ 200 million will cover two in three children in Q4. Reaching children in Q5 will be extremely difficult and expensive, and US$ 250 million will only cover about one third of the children in this quintile (**Figure 6**).

The upper right quadrant graph takes into account that the effectiveness of the same intervention will vary in different quintiles. This is because the quality of delivery usually decreases in the poorest equity strata, making the implementation in Q1-Q3 more effective than in Q4-Q5 (**Figure 6**).

The lower right quadrant graph takes into account that the burden of child deaths is not evenly distributed among the five quintiles and it quantifies the number of deaths averted. It is apparent that removing 50% of the mortality burden in Q1 or Q2 removes similar number of deaths (in absolute terms) as preventing 15% of deaths in Q5 (**see** **Figure 6c**).

Finally, the lower left quadrant graph brings the number averted deaths back to the relationship with the initial investment in US$. This allows us to compare many different scenarios and make informed predictions of cost-effectiveness of each scenario relative to alternative ones – all of which would be impossible to predict intuitively. Thanks to graphs in **Figure 6**, we can now conclude that an investment of US$ 50 million in coverage of children in Q1 will be more cost-effective than any of the other four scenarios, with investing US$ 250 million in covering children in Q5 being the least cost-effective. Still, an investment of US$ 150 million in Q5 (denoted as Q5′, follow the parallel dotted lines in **Figure 6**) would be substantially more cost-effective than an investment of US$ 200 million in Q4 or 250 million in Q5. This means that it is, in fact, more cost-effective to invest $ 150 million into the coverage of children in the poorest quintile (Q5′) than it would be to invest $ 250 million into the coverage of children in Q5.

## DISCUSSION

The interplay between investments to increase intervention coverage and the “returns” in terms of the number of deaths averted is extremely complex and sometimes counter-intuitive. It is intervention-specific, context-specific, and it depends on several variables that show both linear and nonlinear inter-relationships. All of this should be taken into account when planning investment policies and choosing between the many cost-effective interventions at the national and sub-national level. The lines in the “efficiency”, “effectiveness” and “impact” graphs (**Figures 5**** and** **6**) necessarily determine the resulting line in the “cost-effectiveness” graph. Any increase in efficiency and quality of intervention delivery, effectiveness of intervention, or burden of disease within any quintile will improve cost-effectiveness. Looking at **Figures 5**** and ****6**, shows that any rotation of the lines in the “efficiency”, “effectiveness” and “impact” graphs in the clock-wise direction will lead to rotation of the corresponding line in the “cost-effectiveness” graph in the anti-clockwise direction, which is desirable.

**Figures 5**** and ****6** also expose some unexpected and counter-intuitive properties of this framework. First, when lines anywhere in the graph are located counter-clockwise from the line determined with an equation *x = y*, then the cost-effectiveness will decrease with increasing investment in the same population stratum. This means that smaller investments in the same quintiles may prove to be more cost-effective than larger investments. However, if it is possible to change the slope of the lines through improving contexts, then a scenario may be envisaged in which increasing investments in a population quintile also become increasingly cost-effective. This is particularly important for the poorest quintile, as shown in example in **Figure 6**. The scenario presented in both **Figure 5** and **Figure 6** has also shown that in some contexts the most equitable strategy (ie, investing in the poorest quintiles) is not necessarily the most cost-effective. In this case, the decision-making process becomes really difficult, as it cannot be based on any rational framework, but it rather needs to include value choices. When faced with such an interplay of the key determinants in their particular context, policy makers need to decide whether the majority of the society would value improved equity or cost-effective mortality burden reduction (ie, more deaths averted per money invested, irrespective of the increasing inequity) as the more important goal.

Given the level of general interest in tools that could translate accumulated evidence and information into health policy at the national level, and also in improving equity within low and middle-income countries, there is remarkably little evidence on the differential cost of intervention scale-up, effectiveness of intervention, or the composition of mortality burden by equity strata to support even the most basic analysis. With recent progress in assembling information relevant for international child health policy [1-10], we believe that we will soon begin to have sufficient information to develop a model that could allow early comparative analysis, such as the one described above, at the national level in several representative countries. This model should enable the development of guidelines for prioritizing of interventions in different contexts to maximize the reduction in maternal and child mortality burden relative to the funding available, while taking into account the resulting impact on equity.

This model should not be considered in isolation from the other worthy and commendable efforts, all of which have “burden of disease/cost effectiveness analysis” as their essential component, such as those promoted by the Disease Control Priorities Project (DCPP) [19]. For example, the Marginal Budgeting for Bottlenecks (MBB) tool was developed by UNICEF and The World Bank [20], WHO-CHOICE (Choosing Interventions that are Cost-Effective) was developed by the World Health Organization [21], and Lives Saved Tool (LiST) developed by Johns Hopkins University scientists and the Futures Institute [22]. The DCPP authors correctly note that factors other than cost-effectiveness influence priority setting in the real world, so the available evidence has to be considered in the context of local realities [12,19]. Both MBB and WHO-CHOICE provide appropriate contextualization tools. However, the LiST software goes further than other existing tools in several dimensions [12]. LiST contains an expansive evidence base of context-specific intervention effectiveness, generated by researchers from the WHO/UNICEF's Child Health Epidemiology Reference Group (CHERG) [23]. It enables estimation of intervention impact on child mortality at national, regional, and global levels [24,25]. Further important advantages of LiST include its validation in both African and South Asian contexts [26], an ability to perform very specific comparisons between alternative investment strategies over a specified time frame in terms of child survival outcomes [24,25], and its attempt to apply an equity lens [27]. However, due to the gap in information on the key determinants of the interplay between cost-effectiveness and equitable impact in maternal and child mortality reduction, none of the present versions of the available tools allow planning of an equitable strategy to reduce maternal and child mortality.

### Conclusion

In order to assess cost-effectiveness at the national and local level, policy makers would need to know: (i) what is the differential cost of intervention delivery to achieve full coverage in Q1-Q5?; (ii) what is the difference in effectiveness of this new intervention in Q1-Q5?; (iii) what is the difference in mortality burden between Q1-Q5? The interplay among those key determinants needs to be understood, and relative trade-offs need to be quantified before investment decisions can be made. However, in most contexts and for most available interventions there is simply no information on differential cost of scale-up, differential effectiveness and differential mortality burden by equity strata.

We hereby propose a framework that exposes the most important determinants of cost-effectiveness and equitable impact in maternal and child mortality reduction and their interplay. One of the values of this framework is in suggesting how to make interventions delivered to the poorest in the population (Q5) more cost-effective, which is primarily by increasing the efficiency and the quality of intervention delivery, while improving access and promoting care-seeking behaviour and infrastructure to support delivery mechanisms to Q5. The framework also exposes large gaps in information required to understand the interplay between the key determinants - above all, differential cost of intervention delivery by equity strata; differential effectiveness of intervention by equity strata; and differential size and cause composition of mortality burden by equity strata. Finally, the proposed framework should enable modelling of the “thresholds of cost-effectiveness” for the poorest in the population, by starting the analysis from the bottom-left quadrant (“cost-effectiveness graph”) with setting the desired level of cost-effectiveness and, given the burden of mortality, finding the values of effectiveness and cost of scale up that would be required to make the implementation cost-effective while improving equity in the population.
